# Editorial: Break the mental health stigma: bipolar disorder

**DOI:** 10.3389/fpsyt.2024.1377109

**Published:** 2024-02-09

**Authors:** Arghya Pal, Christoph Born, Alfredo B. Cuellar-Barboza

**Affiliations:** ^1^ Department of Psychiatry, All India Institute of Medical Sciences, Raebareli, Uttar Pradesh, India; ^2^ Department of Psychiatry, Psychiatric Hospital Schwäbisch-Hall, Schwäbisch Hall, Germany; ^3^ Department of Psychiatry, Paracelsus Medical University, Nuremberg, Germany; ^4^ Department of Psychiatry, Autonomous University of Nuevo Leon, San Nicolas de los Garza, Mexico

**Keywords:** stigma, bipolar disorder, affective disorder, mania, depression

Bipolar disorder is an episodic illness characterised by multiple mood episodes that includes at least one manic or hypomanic episode and is mostly also accompanied by multiple depressive episodes. However, bipolar disorder represents a complex disorder with a heterogenous phenomenological manifestation. Classically this disorder has an episodic course with the inter-episodic period often marred with subtle residual deficits, often afflicting the most productive years of the patients’ life. This leads to significant dysfunction in the socio-occupational aspects of the patient not only during the episodes but also during the period of remission. The management of the disorder also remains a challenging prospect since it requires skilful reconstruction of the past history and also an anticipation of the further course which governs the choice of pharmacological and non-pharmacological interventions. Thus, this disorder is also considered to be a severe mental illness. Owing to this complex nature of the illness, patients with bipolar disorder experience substantial stigma, in all its forms.

Self-stigma reflects an internalised phenomena where patients endorse certain stereotypes that are labelled on them and that leads to restriction in their community participation, finally culminating in discriminatory behaviour. The process of enacted stigma (which is inflicted by the society) is also inter-related to self-stigma and both these phenomena usually concatenate to jeopardise the patient’s life. It is well established that stigma is ubiquitous in patients of bipolar disorder and that also leads to several further dysfunctions like community participation, decline in self-esteem and quality of life. Research has shown that cognitive dysfunction plays a significant role in the evolution of the chain of events leading up to stigma and there is a need to have a deeper understanding about it. This will also enable us to develop better interventions for the various forms of stigma. The current Research Topic was thus entitled as “*Break the Mental health Stigma: Bipolar Disorder*”. The aim of this Research Topic was to traverse beyond what is already known about stigma in bipolar disorder and to gather knowledge so as to enable us to tailor targeted interventions to stigma.

Wang et al. in their study compared newly diagnosed patients with bipolar disorders. They had classified their recruited subjects into two groups; one with mania as the index episode and other with depression as the index episode. The patients were evaluated with standardised tools to assess cognitive functions and the results were also compared with a control group. The study gave critical hints at attributes that can contribute to stigma. It was found that patients with bipolar disorder had a lower educational level as compared to the control group. The control group also performed better in the cognitive tests as compared to the bipolar disorder group. When the intra-group comparisons were carried out in the patients with bipolar disorder, it was found that patients with depression have more severe cognitive deficits than the bipolar group. This study also found correlations between the socio-demographic parameters with the cognitive deficits. In the next study Quinlivan et al. further provided critical insight into the conundrum of cognitive deficits in patients with bipolar disorder. The authors here compared the executive functioning and self-reported cognitive impairment between patients with bipolar disorder and healthy controls. Interestingly the authors found that though there were no significant difference in the executive functioning of the two groups, the self-reported cognitive impairment was significantly poorer in the bipolar disorder group and it also showed association with the self-esteem of the patients. Both these studies highlighted critical aspects of the cognitive deficits in patients with bipolar disorder and will be instrumental in providing building blocks for anti-stigma interventions in this disorder.

Gardea-Resendez et al. in their study provided insight into how racial background of patients can play a role in the course and management of bipolar disorder. In their study, they retrieved data of 205 patients from a data registry (Rochester Epidemiology Project) who either presented with first episode mania or first episode psychosis and compared their pathways to care and clinical events based on their racial background, which the authors chose to classify as white versus non-white. It was found that the patients hailing from non-white racial background tended to have shorter psychiatric antecedents and older age at the time of first visit to a psychiatric facility. These study goes on to highlight the importance of customising the treatment approaches according to the patient attributes and preferences, which is often governed by these issues. In the fourth and final article of our Research Topic, Mejri et al. provided deep insights into how personal religious beliefs has deep-rooted implications in the disease process. In this study, the authors found that significant proportion of patients with bipolar disorder had to alter their religious practices due to the illness and that led to negative affective states and increased the propensity of experiencing stigma. Stigma in this study could be ascribed to both personal feelings of inadequacy and also critical comments made by others. This study goes on to highlight the importance of customising treatment according to socio-cultural milieu of the patients.

As guest editors of this Research Topic, we found this exercise highly rewarding and we compliment all the authors on successful completion of their work. We believe that this Research Topic has successfully delved into the less studied areas of stigma in bipolar disorder and will open newer research pathways in this topic.

## Author contributions

AP: Writing – original draft, Writing – review & editing. CB: Writing – original draft, Writing – review & editing. AC-B: Writing – original draft, Writing – review & editing.

